# OD48 Improving Public Understanding Of Health Technology Assessment Decisions

**DOI:** 10.1017/S0266462324001740

**Published:** 2025-01-07

**Authors:** Kate Russell, Jackie McCormack, Jennifer Dickson, Daniel Cairns

## Abstract

**Introduction:**

Since 2018, the Scottish Medicines Consortium (SMC) has published a plain English summary for each health technology assessment (HTA) decision called Decision Explained (DE). This provides clear information to patients, Patient Group Partners (PGPs), and the public about what SMC’s decisions mean for them. The DE document was evaluated and updated in 2023 to ensure it continues to meet this purpose.

**Methods:**

The evaluation consisted of two components. All (186) registered PGPs were invited to complete an online five-question survey focusing on the use of the DE document. This included readership, language, and SMC decision-making transparency. A separate focus group for public representatives took place, discussing design, accessibility, and content. Identical sample DE documents were used for both groups for consistency. Survey responses were collated and analyzed. Focus group responses were analyzed using thematic analysis. The DE document was revised, and the new version was considered for implementation by SMC’s Public Involvement Network Advisory Group.

**Results:**

Survey respondents (n=20) found the DE document helpful or very helpful in improving understanding of SMC advice. Some commented on complex language and information about how the medicine works being irrelevant. The focus group commented on excess information and favored simplified content and structure. Analysis of both sets of research data resulted in several recommended changes. These included the decision statement being moved to the start of the document, the language being simplified, and the section on how a medicine works being removed. Revised documents including these changes were prepared and were reviewed and approved by the SMC’s Public Involvement Network Advisory Group.

**Conclusions:**

Published plain English explanations are helpful for improving patient and public understanding of HTA decisions for new medicines. It is important this information is concise, relevant, and aligned to accessibility good practice. The recent review of the SMC DE documents led to changes that help to ensure they meet the needs of stakeholders.

